# Stage of cancer diagnoses among migrants from the former Soviet Union in comparison to the German population – are diagnoses among migrants delayed?

**DOI:** 10.1186/s12889-018-5046-0

**Published:** 2018-01-17

**Authors:** An Bin Cho, Philipp Jaehn, Bernd Holleczek, Heiko Becher, Volker Winkler

**Affiliations:** 1 Institute of Public Health, University of Heidelberg, Im Neuenheimer Feld 324, 69120 Heidelberg, Germany; 2 Saarland Cancer Registry, Saarbrücken, Präsident-Baltz-Straße 5, 66119 Saarbrücken, Germany; 3 Institut für Med. Biometrie und Epidemiologie, Universitätsklinikum Hamburg-Eppendorf, Martinistraße 52, D-20246 Hamburg, Germany

**Keywords:** Lung cancer, Breast cancer, Colorectal cancer, Prostate cancer, Skin cancer, Stomach cancer, Migrants, Stage at diagnosis, Screening, Epidemiology

## Abstract

**Background:**

In this study, we compared stage at diagnosis, standardized incidence ratio (SIR) and standardized mortality ratio (SMR) of most frequent cancer diagnoses between re-settlers (Aussiedler) from the former Soviet Union and the general population in the Saarland in Germany to assess possible delays in diagnosis of cancer among this migrant group.

**Methods:**

Lung cancer, colorectal cancer, breast cancer, prostate cancer, malignant melanoma of the skin and stomach cancer diagnoses among a cohort of 18,619 re-settlers living in the Saarland between 1990 and 2009 were identified by the federal state’s cancer registry. Vital status was available for the respective time-period and used to calculate SIR and SMR in comparison to the autochthonous population. Tumor stages were condensed into local and advanced stages. Odds ratios (OR) for an advanced tumor stage were modeled in dependence of re-settler-status and relevant covariates by logistic regression. Missing values were addressed in a sensitivity analysis. The influence of duration of stay in Germany on advanced stage diagnosis was analyzed among re-settlers.

**Results:**

SIR and SMR of lung and breast cancer were lower among female re-settlers, while SIR and SMR of colorectal and prostate cancer were lower among male re-settlers. SIR and SMR of stomach cancer were elevated among both sexes. Female re-settlers showed an elevated OR for being diagnosed with advanced stage breast cancer. Both male and female re-settlers showed an elevated OR when observing all six sites combined (OR among males 1.47, *p* = 0.04; OR among females 1.37, *p* = 0.05). The result of elevated ORs was supported in the sensitivity analysis. Finally, male re-settlers showed a weak association between duration of stay in Germany and reduced risk for advanced stage diagnosis.

**Conclusion:**

Re-settlers were more likely to be diagnosed at an advanced tumor stage. These findings are in line with previous research having shown unfavorable health care utilization of re-settlers. Overall, low mortality rates despite an increased risk of advanced stage at diagnosis argue for a sufficient follow-up care, comparable to the autochthonous population.

## Background

In 2014, every fifth person living in Germany had a foreign background, thereby making Germany a land of immigration [1]. Cancer is the second leading cause of death in Germany [2], and migrant studies are important to provide insight into early detection and follow-up care of cancer among these vulnerable populations [3, 4]. To date, there is no study that investigated tumor stages at time of diagnosis among immigrants in Germany [3].

A special group within the immigrant population in Germany are the so-called re-settlers (“Aussiedler” or “Spätaussiedler” in German), which is the term for ethnic Germans from the former Soviet Union (FSU), whose ancestors had migrated to Russia particularly during the 17th and 18th century [5]. With the fall of the Iron Curtain in 1989 and the dissolution of the Soviet Union in 1991, more than two million ethnic Germans immigrated without restrictions by invitation of the German government from the FSU to Germany, mainly between 1990 and 2000 [6, 7]. The FSU states, where the vast majority of re-settlers originate from, are Kazakhstan (approx. 50%), the Russian Federation (approx. 30%) and Kirgizstan (approx. 5%) [8]. All re-settlers obtained the German citizenship with all rights and duties directly after migration to Germany [8].

The cancer sites examined in this study are lung cancer (LC), colorectal cancer (CRC), breast cancer (BC), prostate cancer (PC), malignant melanoma of the skin (MM) and stomach cancer (SC), representing the most common cancer sites among re-settlers. In our previous research, we set up a cohort of re-settlers in the federal state of the Saarland, which is located in the south-west of Germany at the French border to study cancer incidence. All-cause cancer mortality of the general population of the Saarland is relatively high compared to Germany [9].

PC, BC, LC and CRC are the most common tumors in the general population of Germany. PC is the most frequent cancer among men in Germany (in 2012 age-standardized incidence rate (ASIR) 106.7 using European standard population) and the third most common cancer related cause of death among men. Similarly, BC is the most frequent cancer found among women and one out of eight women is estimated to develop BC during her lifetime (in 2012 ASIR 117.4) [10]. LC is the second most common cancer and the most common cause of death related to cancer in men (in 2012 ASIR 59.1) with an increasing incidence among women in Germany in the past decade (in 2012 ASIR 27.7) [10]. CRC is the second most common cancer in Germany with every seventh of all cancers diagnosed being either located in the colon or the rectum (in 2012 ASIR for men 57.0, ASIR for women 36.5).

In Germany, cancer screening programs for BC, CRC, PC and MM are being offered systematically for all members of statutory health insurance (SHI) and private health insurance (PHI) on a voluntary basis, while screening for LC is being discussed for cost-effectiveness [11, 12]. The mammography screening of BC was implemented in the Saarland in 2005, inviting women between the ages of 50 and 69 every second year. Most BC cases are diagnosed at an early stage with tumor sizes smaller than 2 cm accounting for 53% of all diagnoses. Additionally, mammography screening as a method of detection was shown to be an independent favorable prognostic factor for overall survival [13]. Considering secondary prevention of CRC, men and women between 50 and 54 are entitled to an annual fecal occult blood test and from the age of 55 onwards to a colonoscopy every 10 years. Following the implementation of screening colonoscopy in Germany in 2002, age-standardized rates for both incidence and mortality significantly declined within a decade, contrary to the increasing trend of incidence rates in the preceding decades. Also, diagnoses of small tumors increased, while tumors with advanced and missing T status decreased from 2000 to 2008 [14]. Screening for PC entails an annual physical examination including a digital rectal exam for men above 45 and is recommended since 1982 [15]. Three quarters of all diagnoses are early stage diagnoses [12]. Finally, since the implementation of the skin cancer screening program in Germany in 2008, MM incidence in Germany has more than tripled compared to the 1980s (ASIR 19.2 in both sexes in 2012), while mortality rates remain unchanged. Two thirds of all diagnoses are early stage diagnoses [12].

To date, there are no screening methods implemented into clinical routine for LC and SC. Almost 70% of all LC patients are diagnosed at stages III or IV [16] and only 25% of all patients are candidates for surgery at the time of diagnosis, showing the limited possibilities for effective treatment at an advanced stage at diagnosis [17]. While the incidence rates for SC have been falling in Germany and other industrial countries for the last few decades (ASIR for men 15.6, ASIR for women 8.3 in 2012), overall prognosis of SC is still unfavorable with relative 5-year survival rates around 30% for both sexes. Two thirds of all diagnoses are in an advanced stage at the time of diagnosis.

Stage at diagnosis is an indicator for the time of diagnosis and an important determinant for overall prognosis. Cancer incidence and mortality show the actual disease burden and the effectiveness of follow-up therapy. Due to varying risk behavior and health beliefs, migrants often represent a vulnerable subpopulation with reduced health care access and lower participation in health care programs, leading to delay in diagnosis [18].

In this study, we will assess whether tumor diagnoses among re-settlers are delayed by comparing the tumor stages at diagnosis between re-settlers and the autochthonous Saarland population utilizing data from the Saarland cancer registry. The comparison of SIR and standardized mortality ratios (SMR) will contribute evidence to the evaluation of follow-up care of re-settlers and whether it is comparable to the general population in Germany.

## Methods

### Data and populations

This retrospective, population-based cohort study observed re-settlers from the FSU who migrated to the federal state of the Saarland during the period 1990 through 2005. The study cohort was built-up by collecting records of immigrated re-settlers from regional population registries and finally included 18,619 re-settlers, which depicts 64% of all re-settlers having settled in the Saarland in this period [19]. In this manuscript the terms general population or autochthonous population of the Saarland describe all inhabitants of the federal state excluding the population of our cohort. The representativeness of the cohort for this period has been ensured in previous studies as cardio-vascular disease mortality is similar in a large representative cohort of re-settlers in the state of Northrhine-Westphalia [19]. Vital status and causes of death of re-settlers was Germany-wide ascertained through local population and health registries between 1990 and 2009 for 95% of the cohort [20].

The Saarland Cancer Registry provided information on incident cases of LC (ICD-9: 162), CRC (ICD-9: 153, 154), BC (ICD-9: 174), PC (ICD-9: 185), MM (ICD-9: 172) and SC (ICD-9: 151) from 1990 to 2009. All cancer diagnoses occurring after an initial cancer diagnosis were considered multiple primary tumors. Multiple primary tumors were included in all analyses. The Saarland Cancer Registry was established in 1967 and covers the population of the federal state with its approximately 1.02 million inhabitants by the end of 2009 [21]. The cohort was set up in the Saarland because of high data quality of the state’s cancer registry.

Information on International Classification of Diseases 9 (ICD-9) code, sex, age at diagnosis, region of residence and TNM classification were available. A variable ‘population density of region of residence’ was created by categorizing each of the seven districts of the Saarland into three categories of population density (less than 300 inhabitants per km^2^ [inh./km^2^], 300 inh./km^2^ to 700 inh./km^2^ and more than 700 inh./km^2^). We introduced the variable to be able to control for rural-urban differences which we assumed to influence access to health services, as access might be poorer in rural areas [22]. Computer assisted record linkage was used to identify re-settlers within local population registries and the cancer registry data via name and date of birth [19].

For the analysis of cancer registry data, the European Network of Cancer Registries (ENCR) recommends a condensed coding of stages [23] due to expected missing values in population-wide data and varying versions of the TNM classification. For this reason, N and M status were used to categorize stages into a local (N0 M0 or N0 Mx), advanced (N + M+ or N + Mx or NxM+) or missing stage (NxM0 or NxMx) for all studied cancer sites. The definition of condensed stages and stage distribution is shown in Table 1.

**Table 1:** Description and distribution of condensed staging

Stage	T status	N status	M status	Cancer site	Re-settlers *N* (%)	General population *N* (%)
Local	Any	–	–	Lung cancer	5 (5.3%)	1221 (7.8%)
				Colorectal cancer	9 (11.5%)	3059 (16.1%)
				Breast cancer	31 (33.7%)	5512 (35.0%)
				Prostate cancer	4 (7.5%)	1357 (11.0%)
				Malignant melanoma	12 (57.1%)	1514 (53.9%)
				Stomach cancer	11 (16.9%)	367 (7.5%)
				All sites combined	72 (17.9%)	13,030 (18.5%)
	Any	–	Missing	Lung cancer	1 (1.1%)	436 (2.8%)
				Colorectal cancer	17 (21.8%)	3505 (18.5%)
				Breast cancer	4 (4.3%)	883 (5.6%)
				Prostate cancer	14 (26.4%)	2069 (16.7%)
				Malignant melanoma	1 (4.8%)	39 (1.4%)
				Stomach cancer	3 (4.6%)	358 (7.3%)
				All sites combined	40 (9.9%)	7290 (10.4%)
Advanced	Any	+	–	Lung cancer	22 (23.4%)	2272 (14.6%)
				Colorectal cancer	9 (11.5%)	1862 (9.8%)
				Breast cancer	30 (32.6%)	3579 (22.7%)
				Prostate cancer	0 (0.0%)	110 (0.9%)
				Malignant melanoma	1 (4.8%)	107 (3.8%)
				Stomach cancer	8 (12.3%)	462 (9.4%)
				All sites combined	70 (17.4%)	8392 (11.9%)
	Any	+	Missing	Lung cancer	7 (7.4%)	715 (4.6%)
				Colorectal cancer	15 (19.2%)	2555 (13.5%)
				Breast cancer	9 (9.8%)	1352 (8.6%)
				Prostate cancer	0 (0.0%)	261 (21.1%)
				Malignant melanoma	0 (0.0%)	29 (1.0%)
				Stomach cancer	7 (10.8%)	622 (12.6%)
				All sites combined	38 (9.4%)	5534 (7.9%)
	Any	Any	+	Lung cancer	33 (35.1%)	5393 (34.6%)
				Colorectal cancer	15 (19.2%)	2861 (15.1%)
				Breast cancer	8 (8.7%)	1115 (7.1%)
				Prostate cancer	1 (1.9%)	605 (4.9%)
				Malignant melanoma	2 (9.5%)	156 (5.6%)
				Stomach cancer	14 (21.5%)	988 (20.1%)
				All sites combined	73 (18.1%)	11,118 (15.8%)
Missing	Any	Missing	–	Lung cancer	1 (1.1%)	322 (2.1%)
				Colorectal cancer	0 (0.0%)	226 (1.2%)
				Breast cancer	0 (0.0%)	312 (2.0%)
				Prostate cancer	0 (0.0%)	572 (4.6%)
				Malignant melanoma	2 (9.5%)	134 (4.8%)
				Stomach cancer	0 (0.0%)	60 (1.2%)
				All sites combined	3 (0.7%)	1626 (2.3%)
	Any	Missing	Missing	Lung cancer	25 (26.6%)	5206 (33.4%)
				Colorectal cancer	13 (16.7%)	4879 (25.8%)
				Breast cancer	10 (10.9%)	2989 (19.0%)
				Prostate cancer	34 (64.2%)	7400 (59.8%)
				Malignant melanoma	3 (14.3%)	830 (29.5%)
				Stomach cancer	22 (33.8%)	2063 (41.9%)
				All sites combined	107 (26.6%)	23,367 (33.2%)

(−: not involved, +: involved)

### Statistical methods

Person-years (PY) were calculated for each sex, five-year age group, and calendar year. SMR for each cancer site was calculated using recorded deaths and PY of re-settlers together with age-specific death rates of the Saarland population. For SIR analysis, re-settlers were censored when moving out of the Saarland and PY were calculated for living within the Saarland accordingly. Incident cancer diagnoses among re-settlers, PY within the Saarland and age-specific incidence rates of the Saarland population were used to calculate SIR. SIR was calculated for each tumor type separately and for all sites combined. Ninety-five percent confidence limits (95%CI) were calculated using the exact method [24].

Logistic regression was performed to assess the association between advanced stage at diagnosis and re-settler status. Condensed stage (advanced-local) at diagnosis was the dependent, and re-settler status the independent variable while adjusting for age at diagnosis, year of diagnosis and population density of region of residence. The model was calculated separately for each sex and cancer site as well as for all sites combined including all LC, CRC, BC, PC, MM and SC diagnoses from the Saarland cancer registry between 1990 and 2009. Male MM cases were not analyzed individually, as there were no advanced stage diagnoses registered among re-settlers, but they were included in the combined analysis.

A sensitivity analysis including all studied cancer sites was performed to assess the influence of cases with missing information on staging, in which two possible scenarios were considered. In the best-case scenario, all cases with missing stage were considered as local stage and in the worst-case scenario as advanced stage.

An additional logistic regression was performed among re-settlers only to assess the influence of duration of stay in Germany on the diagnosis of advanced stages. As we assumed a non-linear trend, condensed stage at diagnosis was modeled conditional to duration of stay in Germany, transformed as a quadratic term. Transformation was determined using fractional polynomials [25]. The analysis was adjusted for age at diagnosis, year of diagnosis and population density of region of residence. We calculated this model separately for each sex and for the six cancer sites combined.

The statistical software R was used to conduct statistical analyses (Version 3.13).

## Results

The Saarland Cancer Registry observed 15,565 diagnoses of lung cancer, 18,947 diagnoses of colorectal cancer, 15,742 diagnoses of female breast cancer, 12,374 diagnoses of prostate cancer, 2809 diagnoses of malignant melanoma and 4920 diagnoses of stomach cancer in the Saarland between 1990 and 2009. Among the re-settler cohort 94, 78, 92, 53, 21 and 65 diagnoses were observed, respectively.

A descriptive analysis of the re-settler cohort including their accumulated PY used to calculate SIR and SMR is shown in Table 2. Concerning LC, male re-settlers’ SIR and SMR was somewhat higher than the general population of the Saarland however not significantly, while female re-settlers had significantly lower SIR and SMR of LC (Table 3, SIR = 0.42 95%CI 0.21–0.76, SMR = 0.53 95%CI 0.29–0.89). Male re-settlers had lower SIR and SMR of CRC (SIR = 0.50 95%CI 0.34–0.72, SMR = 0.52 95%CI 0.30–0.85), while female re-settlers had comparable SIR and SMR. Female re-settlers had lower SIR and SMR of BC (SIR = 0.80 95%CI 0.65–0.98, SMR = 0.63 95%CI 0.42–0.93), while male re-settlers had lower SIR and SMR of PC (SIR = 0.75 95%CI 0.57–0.98, SMR = 0.30 95%CI 0.11–0.65). Both male and female re-settlers had comparable SIR and SMR of MM. Considering SC, both male and female re-settlers had higher SIR and SMR (SIR for males = 2.22 95%CI 1.55–3.07, SMR for males = 1.91 95%CI 1.25–2.81, SIR for females = 2.32 95%CI 1.56–3.35, SMR for females = 2.15 95%CI 1.38–3.18). Finally, for all cancer sites combined, male re-settlers had comparable SIR and SMR and female re-settlers had somewhat lower SIR and SMR however not significantly. Additionally, SMR was comparable to SIR in all cases.

**Table 2:** Descriptive results of the re-settler cohort for the follow-up period 1990–2009

Number of cohort members	18,619	
Male	8976 (48.2%)	
Female	9643 (51.8%)	
Immigration period	1990–2005	
1990–1993	6933	
1994–1997	6536	
1998–2005	5150	
End of follow-up		
Alive	16,391 (88.0%)	
Dead	1314 (7.1%)	
Cause of death known	1204 (91.6%)	
Lost to follow up	914 (4.9%)	
	Incidence (inside Saarland)	Mortality (Germany-wide)
Person-years	204,366.3	243,369.1
Males	98,622.6	117,310.7
Females	105,743.7	126,059.1

**Table 3:** Standardized incidence ratios and standardized mortality ratios

Tumor type	Males				Females			
(ICD-10)	Observed		SIR	SMR	Observed		SIR	SMR
	Incidence	Mortality	(95% CI)	(95% CI)	Incidence	Mortality	(95% CI)	(95% CI)
Lung (C33–34)	83	87	1.16 (0.94–1.44)	1.09 (0.89–1.35)	11	14	0.42 (0.21–0.76)	0.53 (0.29–0.89)
Colorectal (C17–21)	29	16	0.50 (0.34–0.72)	0.52 (0.30–0.85)	49	27	0.92 (0.68–1.22)	0.91 (0.60–1.33)
Breast (C50)					92	27	0.80 (0.65–0.98)	0.63 (0.42–0.93)
Prostate (C61)	53	6	0.75 (0.57–0.98)	0.30 (0.11–0.65)				
Malignant melanoma (C43)	9	2	0.89 (0.41–1.69)	0.73 (0.09–2.64)	12	2	1.00 (0.52–1.76)	0.86 (0.10–3.11)
Stomach (C16)	36	26	2.22 (1.55–3.07)	1.91 (1.25–2.81)	29	24	2.32 (1.56–3.35)	2.15 (1.38–3.18)

Results of the logistic regression of condensed staging are shown in Table 4. Both male and female re-settlers were more likely to be diagnosed at an advanced condensed stage than the general population, yielding an adjusted odds ratio (OR) of 1.47 (95%CI 1.02–2.10) for males and 1.37 (95%CI 1.00–1.89) for females when analyzing all six sites combined. Male re-settlers had elevated ORs for advanced stage diagnosis of LC and CRC, although not significantly. Female re-settlers showed a higher percentage distribution of advanced stage at diagnosis of BC with an adjusted OR of 1.60 (95%CI 1.03–2.48) and of MM with an adjusted OR of 4.29 (95%CI 0.99–18.60). Female re-settlers also had increased ORs in LC and CRC cases. Re-settlers had a reduced OR of advanced stage diagnosis of SC and PC cases, however not significantly. In the sensitivity analysis, re-settler status was associated with an advanced stage at diagnosis for both sexes in the best-case scenario with adjusted ORs of 1.32 (95%CI 1.00–1.75) for male and 1.44 (95%CI 1.08–1.91) for female re-settlers. Re-settlers also presented elevated ORs in the worst-case scenario, however not significantly. Re-settlers had significantly less missing data on condensed staging than the general population (*p* < 0.01).

**Table 4:** Logistic regression of advanced stage diagnosis in dependence of re-settler status, adjusted for possible confounders

Tumor type	Males		Females	
	Odds ratio (95% CI)	*p*-value	Odds ratio (95% CI)	*p*-value
Lung cancer	2.05 (0.82–5.12)	0.13	1.48 (0.18–12.12)	0.71
Colorectal cancer	1.47 (0.64–3.53)	0.37	1.24 (0.66–2.31)	0.50
Breast cancer			1.60 (1.03–2.48)	0.04
Prostate cancer	0.24 (0.03–1.84)	0.17		
Malignant melanoma of the skin	NA^a^	NA^a^	4.29 (0.99–18.60)	0.05
Stomach cancer	0.77 (0.29–2.04)	0.60	0.54 (0.21–1.39)	0.20
All sites combined	1.47 (1.02–2.10)	0.04	1.37 (1.00–1.89)	0.05
Sensitivity analysis				
Best case scenario (all missings as local)	1.32 (1.00–1.75)	0.05	1.44 (1.08–1.91)	0.01
Worst case scenario (all missings as advanced)	1.32 (0.95–1.84)	0.09	1.22 (0.90–1.65)	0.20

^a^not applicable due to low number of cases

There was a quadratic association (*p* = 0.10) between the duration of stay (years) in Germany and a reduced risk of an advanced stage at diagnosis in male re-settlers (Table 5). For female re-settlers, no association was found.

**Table 5:** Association between duration of stay in Germany and advanced stage diagnosis in re-settlers

	Males		Females	
Coefficients	Odds ratio	*p*-value	Odds ratio	*p*-value
Duration of stay (years^2^)	0.995	0.10	1.002	0.37
Age at diagnosis (years)	0.963	0.04	0.996	0.77
Year of diagnosis^a^	1.113	0.07	0.894	0.02
Population density of region of residence [inhabitants/km^2^]		0.26		0.75
< 300 (ref.)				
300–700	0.169		0.734	
> 700	0.173		0.705	

^a^coded as calendaryear-1990

## Discussion

Both male and female re-settlers were more likely to be diagnosed at an advanced stage compared to the general population in the Saarland, as seen for all studied cancer sites combined among both sexes and female BC cases. However, they had lower or comparable cancer incidence and mortality rates of all studied cancer sites except SC. In general, cancer incidence and mortality reflected rates in their country of origin [26]. Additionally, there was a weak association of duration of stay in Germany and reduced risk of advanced stage at diagnosis among male re-settlers, while no association was found among female re-settlers.

The main strength of this study was its representativeness with an observation period of 20 years and its inclusion of a large number of re-settlers who are generally hard to identify in population health research [19]. Additionally, the Saarland Cancer Registry provides a high completeness of cover of about 95%, as cancer registration is mandatory by law [27]. In addition, there have not been any studies about the stage at diagnosis of cancers among migrant subpopulations in Germany before [3].

One restriction of this study was its incomplete distinction of re-settlers and non-re-settlers. Since the study cohort covered only 64% of all re-settlers living in the Saarland, incidence data of the general population of the Saarland included a small number of re-settler cases. A complete distinction between re-settlers and the general population would have resulted in clearer results. However, results were biased towards the null, which makes us confident of having avoided overestimation in our results.

Numbers of deaths among the re-settler cohort are based on a Germany-wide follow up, which were compared to the mortality rates of the general population in the Saarland in SMR analysis. For most of the follow-up period, the general population of the Saarland had higher male LC mortality rates than the average rate in Germany [19]. In a previous publication on re-settlers, SIR analysis excluded multiple primary tumor diagnoses and the general German population was used as the reference population in SMR analysis [19, 28], accounting for lower SIR and SMR in the present analysis.

In order to unify data that is based on changing versions of the TNM classification over a period of 20 years, data on lymph node involvement (N) and distant metastasis (M) according to the Union for Cancer Control (UICC) was used to determine a condensed staging system as proposed by the European Network of Cancer Registries [23]. N0 Mx was defined as local stage, since 96.5% of all complete cases with a N0 diagnoses in the dataset were local stage (N0 M0) diagnoses. When defining N0 Mx as missing, we obtained similar ORs as shown in Table 4 for all cancer sites combined (OR for men 1.48, 95% CI 0.98–2.23; OR for women 1.43, 95% CI 1.00–2.04), showing that a misclassification bias is unlikely using this classification.

In the sensitivity analysis, re-settlers had elevated ORs in both the best-case and the worst-case scenario, while not significant on a level of α = 5% in the worst-case scenario. This is attributable to the significantly lower proportion of missing data in the staging variable of re-settlers compared to the general population (*p* < 0.01). Female re-settlers had less death certificate only (DCO) cases than the general population (*p* = 0.04), while DCO distribution was comparable among male re-settlers. Additionally, the confidence interval marginally included 1.00 in both sexes in the worst-case scenario.

Significant greater completeness of data among re-settlers suggests differences in the process of diagnoses between re-settlers and non-re-settlers. It is difficult to predict whether the missing data in the staging variable is more likely to be local or advanced. However, we are confident that the results of the sensitivity analysis support our findings of an increased risk of advanced stage diagnoses among re-settlers despite missing data.

Population density of region of residence was used to adjust for assumed rural-urban differences in access to health care. We hypothesized that people living in rural areas might have worse access to health care and thus might present more advanced stages at diagnosis than people living in urban areas [22, 29]. Also, the age and the year of diagnosis were considered possible confounders for the analysis of stage at diagnosis.

Somewhat higher incidence and mortality from LC among male re-settlers can be best explained with tobacco smoking. An analysis of micro census data supports this conclusion by having shown that especially male re-settlers import health-related risks such as a high smoking prevalence from their countries of origin (Kazakhstan, Russia, Kirgizstan), while prevalence converges with increasing length of stay to the prevalence of the country of destination [30]. The finding of a lower LC incidence among female re-settlers is in line with studies having demonstrated a lower smoking prevalence in this population [30]. Current and previous smokers are more likely to be diagnosed with advanced stage LC than never-smokers, which could have contributed to our finding of an insignificantly higher proportion of advanced stages among male re-settlers with LC [31]. Additionally, early symptoms of LC are very unspecific, emphasizing the importance of an early involvement of specialists in the diagnostic process of suspected LC patients [16]. Recent analyses show that re-settlers may be less likely to visit specialists [32, 33], which thus might contribute to a possible delay in LC diagnosis among re-settlers.

Lower BC incidence and mortality of female re-settlers reflect rates in the Russian Federation [26]. This may be due to parallels of reproductive factors in their countries of origin. Early parity is a protective factor for BC [34] and women in the Russian Federation are on average 4–5 years younger at their first birth than women in Germany [26, 35]. Furthermore, advanced stage is associated with patient delay of diagnosis and non-participation in general health screening examinations for BC in the general population of the Saarland, suggesting a need for better inclusion of female re-settlers into the German healthcare system [36]. However, BC screening was introduced only in 2005. With an observation period of our cohort from 1990 to 2009, we assume that the influence of screening on cancer stages in our cohort was probably small. Additionally, a lower mortality despite more advanced stages argues for an efficient follow-up care.

Elevated risks of stomach cancer and reduced risks of colorectal and prostate cancer are in line with previous findings of increased risks of cancers related to infections in early life (e. g. stomach cancer) and reduced risks of cancers related to a western lifestyle (e. g. colorectal cancer, prostate cancer) among re-settlers. The differences of mortality also reflect rates in the Russian Federation [26]. These differences may be due to various risk factors including lifestyle, diet and *Helicobacter pylori* infection [37].

The comparison of the Gleason score may have been more informative than the comparison of condensed stages in the case of PC [38], however, this data was not available to us. A large proportion of cases with missing data on staging particularly among PC and to a smaller degree also among SC cases makes it difficult to conclude on differences of those entities between re-settlers and the general population. Concerning SC, cases were frequently detected at a final stage also in the general population of the Saarland, which decreased the difference to re-settlers.

CRC screening has been introduced in Germany in 2002, thus, re-settlers have had access to regular colonoscopy examination during the last 8 years of observation. As stage at diagnosis was not more advanced among re-settlers, we could not find strong evidence for major differences in the diagnostic process. However, more research is needed to draw a final conclusion on possible delays in the diagnosis of CRC.

Interestingly, there was a distinct disparity in the distribution of stages between male and female re-settlers with MM. While female re-settlers showed a more balanced distribution of stages and an elevated risk for an advanced stage at diagnosis compared to the autochthonous population, male re-settlers presented no advanced stage diagnoses and 8 local stage diagnoses. Due to this imbalanced distribution, logistic regression could not be carried out for male MM cases. Furthermore, MM screening could not have played a significant role in the diagnostic process as it was introduced only at the end of the observation period. Ultimately, no clear conclusion can be drawn due to the small sample size for MM. Considering our finding of later stages of MM cases among female re-settlers, we propose to explore differences in MM screening behavior between the sexes in the future. Overall, SMR and SIR of re-settlers were comparable and SMR was never significantly elevated except for SC. Those results argue for an efficient treatment, comparable to the autochthonous population. Future survival studies investigating re-settlers should adjust for tumor stage at diagnosis.

Comparable mortality rates despite an overall elevated risk of advanced stage at diagnosis may be attributable to several factors besides the diagnostic process. Different histologic subtypes may play a role, as it has been shown in the case of SC, where re-settlers show a higher proportion of prognostic favorable histologic types compared to the general population of the Saarland [37]. In addition, specific cancer characteristics such as speed of growth could lead to more advanced stages in this group.

Our results finally support previous findings on health care utilization of re-settlers. Findings from the KORA survey in 2000 showed that re-settlers are less likely to participate in cancer prevention programs and that they indicate more often being less informed about the health care system in Germany [32]. Also, they were more likely to seek medical advice from the lay referral system (seeking for medical advice online or from family and friends), rather than visiting a specialist physician [39]. Our finding of more advanced stages of several cancer sites with different etiologies among re-settlers is supporting the conclusion that a delay in the diagnostic process rather than the dominance of faster growing cancer entities is a likely explanation for more advanced stages of re-settlers.

All re-settlers obtained the German citizenship with migration to Germany which gave them direct access to German social security systems including health insurance [8]. In general, the ratio of people without access to health coverage is low in Germany. In the year 2007, 2 years before the introduction of mandatory health insurance in 2009, the number of inhabitants without health insurance peaked with 400,000 (0.5%) [40]. In addition, socio-economic position of re-settlers is moderate, being in-between autochthonous Germans and other migrants in Germany [8]. In conclusion, health care utilization rather than health coverage is a plausible explanation for their more advanced stages.

Last, there was a weak association of duration of stay in Germany with the risk of advanced stage at diagnosis of male re-settlers, but not of female re-settlers. With longer duration of stay, the risk for later cancer diagnosis among male re-settlers seems to decrease slowly. Possible reasons include slightly ameliorating integration into the German healthcare system, a change in risk factor exposure or health care utilization [24, 28, 41].

## Conclusion

In conclusion, re-settlers were more likely to be diagnosed at an advanced tumor stage. Findings are most likely due to high proportions of advanced stages of LC cases among males and BC and MM cases among females, while re-settlers presented a comparable proportion of stages at diagnosis of CRC, SC and PC cases. Thus, the tendency to rarely visit specialists and to participate in prevention programs less frequently may lead to a delay in diagnosis. Overall, comparable mortality rates despite an increased risk of advanced stage at diagnosis argue for an efficient follow-up care comparable to the autochthonous population. Future studies should focus on determining specific risk factors for a delay in diagnosis of cancer and attainable preventive methods to promote early diagnosis in a specialist setting for this group of migrants.

## Abbreviations

ASIR: Age-standardized incidence rate
BC: Breast cancer
CI: Confidence limits
CRC: Colorectal cancer
DCO: Death certificate only
ENCR: European Network of Cancer Registries
FSU: Former Soviet Union
ICD: International Classification of Diseases
LC: Lung cancer
MM: Malignant melanoma of skin
OR: Odds ratio
PC: Prostate cancer
PY: Person-years
SC: Stomach cancer
SIR: Standardized incidence ratio
SMR: Standardized mortality ratio
UICC: Union for International Cancer Control
